# When Care Turns Hostile—Threats and Violence Toward Staff in Somatic Healthcare

**DOI:** 10.3390/nursrep16010005

**Published:** 2025-12-24

**Authors:** Anne Karine Østbye Roos, Stine Eileen Torp Løkkeberg, Vigdis Abrahamsen Grøndahl, Ann Karin Helgesen

**Affiliations:** 1 Intensive Care Department, Ostfold Hospital Trust, 1714 Grålum, Norway; anne.karine.ostbye.roos@so-hf.no; 2 Faculty of Health, Welfare and Organization, Ostfold University College, 1757 Halden, Norway; stine.t.lokkeberg@hiof.no (S.E.T.L.); vigdis.a.grondahl@hiof.no (V.A.G.); 3 Department of Health and Care Sciences, Faculty of Health Sciences, UiT The Arctic University of Norway, 9037 Tromsø, Norway

**Keywords:** workplace violence, workplace safety, healthcare personnel, nurses, hospital, incident reporting, qualitative research

## Abstract

**Background/Objectives:** Workplace violence in the healthcare sector is a growing global concern. Defined as incidents where staff are abused, threatened, or assaulted in work-related contexts, this issue affects over half of healthcare personnel worldwide, with nurses being particularly vulnerable. The consequences are far-reaching, leading to diminished service quality, workforce turnover, reduced healthcare access, and increased costs. Despite its physical and psychological impact, workplace violence remains underreported, especially in its psychological forms, which have historically been underestimated. **Methods**: This study applies Per Isdal’s typology of violence to analyze incident reports from a hospital setting. By doing so, it offers a structured framework for understanding the multifaceted nature of workplace violence. By categorizing and examining how different forms of violence co-occur and manifest in daily professional interactions, the study aims to contribute to more systematic documentation and theoretical understanding of the field. **Results:** In total, 247 incidents were analyzed. Physical violence was the most frequently reported type with 167 incidents, followed by psychological violence with 125 cases. Material violence accounted for 28 reports, sexual violence for 10, and latent violence for 4, indicating that physical and psychological aggression dominates the spectrum of workplace violence in this context. **Conclusions**: The prevalence and complexity of violent incidents targeting healthcare personnel highlight the pressing need for actionable policies and evidence-based interventions that prioritize staff safety and psychological well-being. Establishing clear definitions of violence, alongside fostering a culture of reporting, is essential to create safer and more resilient healthcare environments.

## 1. Introduction

The World Health Organization [1] has highlighted workplace violence in the healthcare sector as a global issue with several implications for healthcare personnels. The general definition of workplace violence is as follows: “Incidents where staff are abused, threatened, or assaulted in circumstances related to their work, including commuting to and from work” [1]. These incidents involve an explicit or implicit challenge to an individual’s safety, well-being, or health. Workplace violence in the health sector is increasing due to reforms, work pressure, stress, and social instability [2]. In healthcare, the nursing profession is exposed to the highest risk of being subjected to workplace violence [3,4,5].

Consequences of workplace violence include a decline in service quality, turnover, reduced health services, and increased health costs [1,6]. Exposure to workplace violence has also been linked to lower levels of job satisfaction and reduced occupational performance [3,4,6]. A review by Diez-Canseco [7] indicated that workplace policies and training increase employee’s reporting of sexual harassment. Although consequences of being subjected to workplace violence can be both physically and psychologically damaging [8], studies show that healthcare personnel underreport workplace violence [8,9,10,11,12,13,14]. In previous years, physical violence has consistently been recognized. However, the existence of psychological violence has long been underestimated and is currently emerging as a concern in health-related workplaces [1]. Scandinavian countries exhibit significantly lower prevalence rates compared to other European nations and the United States [6]. However, a national study conducted in Norway in 2023 found that 42.6% of registered nurses had been subjected to workplace violence, underscoring a pressing need for systematic improvements in the psychosocial work environment [15].

Despite increasing recognition of its prevalence, the documentation and analysis of workplace violence often lack theoretical grounding and systematic categorization. This study builds on Per Isdal’s [16] definitions of different forms of violence as a practical tool for analyzing incident reports in a hospital setting. Isdal’s approach offers a theoretically grounded perspective as it conceptualizes violence within relational and systemic contexts. By utilizing this structured approach, the study aims to capture the multifaceted nature of violence and to explore how different forms of violence may co-occur and are expressed in everyday professional interactions.

## 2. Materials and Methods

In the book The Meaning of Violence [16], Isdal identifies five distinct categories of violence: physical, psychological, sexual, material and latent. The different categories of violence are described in Table 1. These categories serve as analytical tools for interpreting staff’s reported experiences of violence and for understanding the complex nature of violent behavior in our material. By applying these definitions, the study aims to systematically classify reported incidents of violence, with a focus on identifying patterns of co-occurrence and the contextual presentation of various forms of violence within the hospital environment.

### 2.1. Study Setting

The research was carried out within a hospital trust in southeastern Norway. The hospital serves a population of around 330,000 people and is regarded as medium-sized by Norwegian standards. Somatic and mental health care units are co-located in the hospital.

### 2.2. Entrance to the Field and Sample

The hospital has an electronic system for reporting incidents. The hospital’s Data Protection Officer and the Director of the Quality Department granted permission to access anonymized information from the system.

### 2.3. The Incident Reporting System

The incident reporting system is an electronic system set up for all hospital staff to report adverse events such as clinical and non-clinical incidents, near misses, and incidents affecting the staff’s health, safety, and environment. When incidents are submitted through the electronic system, this facilitates comprehensive documentation, including detailed information about the event, the individuals involved, and the outcomes. This enables a systematic approach to incidents where different events and causes can be selected electronically. The immediate supervisor at the respective departments is responsible for reviewing reported incidents and categorizing them in accordance with the predefined criteria established within the reporting system. If additional expertise or intervention is required, the case is forwarded to the appropriate department for further action.

By systematically documenting incidents, hospital management can analyze data to detect patterns, determine root causes, and implement corrective actions to prevent future occurrences. The data can also be used for research purposes.

### 2.4. Adverse Events Included in the Study

Initially, the researchers intended to include only incidents involving nurses and other healthcare personnel employed in somatic units. However, after reviewing the material, it was decided to also include incidents reported by security services. The rationale for this inclusion and thereby changing the sampling plan was that in many incidents, the security services were involved in protecting and ensuring the safety of healthcare personnel and participating in environmental measures in patient care. It was therefore considered important and appropriate to include these descriptions to ensure clarity and thoroughness in describing what healthcare personnel, in collaboration with the security services, are subjected to. We aimed to offer a detailed perspective supporting our results by doing so, as well as a more comprehensive representation of different staff categories at risk. Reports were not merged or excluded as duplicates. This methodological decision was made to ensure that all personal narratives were retained, allowing us to explore variations in perception and reporting.

### 2.5. Data Collection

In this study, anonymized data extracted from the hospital’s incident reporting system has been used. Incidents recorded as violence and threat events in the hospital’s incident reporting system over a period of 18 months, from staff working in somatic units during 2023 and 2024, were included. A total of 267 separate incidents were reported as acts of violence and threats. During data collection, we identified several cases that did not meet the inclusion criteria for our study. Specifically, 20 cases were excluded from the analysis because they either lacked sufficient detail to describe relevant incidents or were misclassified by managers as episodes of violence or threats. After excluding irrelevant and incorrectly registered cases, the material therefore consisted of 247 incidents. This refined data set allows for a more accurate and focused analysis of relevant incidents.

### 2.6. Ethical Considerations

The recorded incidents contain detailed personal information about patients and staff, as well as the time and location of the events. To ensure anonymity and confidentiality, the first author was provided with a dataset from the hospital’s Quality Department that safeguarded these aspects. An advisor from the Quality Department generated a report and manually reviewed each case to ensure that personal and location-identifying information was removed. The report sent to the researchers included a description of the incidents, the immediate consequences of the events, any immediate actions taken, and the staff’s suggestions for further measures. No personal information, such as names, age or gender, was included.

To make sure that no ethically inappropriate material was included, the first author also reviewed the material before granting access to the other authors. Despite being anonymized, the description of one specific case was of such nature that it was removed from the sample to make sure that the patient was not compromised. Certain elements within the quotations have been further anonymized to ensure the confidentiality of both patients and staff. As the study did not aim to generate new knowledge about health or disease, the project did not fall under the Norwegian Health Research Act and did not require approval from the Regional Committees for Medical And Health Research Ethics.

The study has been reviewed and approved by the hospital’s Data Protection Officer.

### 2.7. Study Aims

What forms of violence and threats are staff working in somatic hospital units subjected to?

### 2.8. Data Analysis

The hospital’s Quality Department provided the data in a Microsoft Excel sheet (Microsoft ©Excel© for Microsoft 365 MSO (Version 2510 Build 16.0.19328.20266) 64 biters. The analysis followed Braun and Clarke’s [17] approach to thematic analysis, which emphasizes a systematic yet flexible process of identifying and interpreting patterns in qualitative data. To begin, all authors read through the entire material to gain an overall sense of its content. The first author then conducted a detailed, word-by-word review of the text, identifying key sentences and words that captured the nature of the reported incidents.

To structure the material, the first author inserted additional columns into the Excel file and coded various acts of threats and violence as they appeared in the data. In total, 33 different acts were identified. The filtering function in Excel was used to obtain an overview of the frequency of each act, allowing for both qualitative exploration and a degree of quantification.

The analysis began inductively, with codes emerging from the material itself, and later moved toward a deductive phase informed by Isdal’s [16] definitions of violence. This two-step process allowed the team to combine the open exploration of inductive coding with the structured categorization of deductive coding, ensuring that both manifest and more subtle forms of violence were captured.

Finally, all authors collaboratively refined the coding and interpretations, discussing and adjusting the categories until consensus was reached. By doing this, inter-coder reliability was incorporated to enhance the rigor of qualitative analysis by ensuring consistency in the coding framework among researchers. Formal inter-coder reliability measures were not applied, as the study followed a qualitative, consensus-oriented methodology in which coding decisions were established through collaborative discussion rather than quantified statistically [17].

Selected excerpts from the material were included to illustrate the results, strengthen the trustworthiness of the analysis, and exemplify the five categories of violence. Within each category, relevant excerpts were selected to illustrate key results. Selection was guided by the research objectives and aimed to capture both the breadth and depth of perspectives represented in the reported cases. The selection process was refined through multiple systematic reviews to offer consistency and precision.

## 3. Results

The data included both numerical data and text excerpts from selected reports written by healthcare personnel. Table 2 presents the reported types of violence and the corresponding number of incidents associated with each type. The analysis of 247 incidents describes a significant number of violent acts against staff, totaling 334 distinct types. The police were involved in 59 incidents, and security staff in 109.

Overlapping each other, several of the reported cases included more than one type of violent act, with the combination of physical and psychological violence being the most common act of violence. Table 3 illustrates the extent to which different violent incidents overlap and how they occur in combination with one another.

During the analysis of the material, we found variations in how staff reported incidents. Some staff used technical, concrete language, providing precise information without emotional expressions or words. Other staff used descriptions that included emotional expressions that highlighted the effects that violence or threats had on them. The context, setting, and/or situation surrounding the individual incidents were not always described. Some of the incidents were reported by observing colleagues, rather than by the person subjected to workplace violence.

### 3.1. Physical Violence

Of all the cases described, physical violence was the most frequently reported type of incident. The types of physical violence and number of reported incidents are shown in Table 4. In several cases, multiple types of violent incidents were described in a single case. Being hit was the most frequently described type of violent incident. Being squeezed or headbutted was described the least.

These incidents were solely carried out by patients. No relatives, no visitors or other co-workers were involved in the execution of physical violence against staff. In 45 of the cases, staff reported that they suffered physical injuries such as pain in the attacked area, bruised skin, bleeding, or muscular injuries.

In multiple cases, patients were described as displaying very aggressive behavior. The common denominator was often that the patients had conditions like dementia, delirium, were under the influence of drugs or alcohol or were in a state of psychological imbalance. One staff member reported:

*“Patient with dementia. Very agitated. Climbs out of bed, attempts to pull out the catheter. Two security guards were present. When I entered to assist, the patient hit my right forearm. The patient also hits the security guards and has previously bitten another colleague.”*

In several cases, it was described that handling situations requires a significant number of staff. It was also reported that there were not always compulsory measures in place to manage the patient effectively, as one nurse explains:

*“Patient with delirium, violently aggressive towards staff present. The patient kicked, hit, and tried to bite. The patient squeezed a colleague’s hand, struck the colleague and pinned them against the wall. The patient hit me hard and kicked me. The patient also struck a security guard. There are four healthcare personnel who have experienced violence from this patient on this shift as well as one security guard. The patient does not have a compulsory treatment order under the Mental Health Act.”*

The material also provided detailed accounts of incidents in which both fellow patients and healthcare personnel were exposed to serious potential danger. Life-threatening incidents involving weapons were also described, as this doctor reported:

*“I found a patient experiencing severe tremors, cold sweats, and vivid visual hallucinations in the corridor. The patient was reoriented back to bed but later became more agitated, refusing staff assistance and insisting on going outside alone. The patient was eventually found in a double room with two female patients and had to be calmed down in the staff room, the patient then became aggressive and grabbed the staff member by the neck. The patient managed to get hold of scissors and threatened the staff. Security and police were called, and they managed to de-escalate the situation.”*

The security services were involved in 65 of the total 167 incidents coded as physical violence, while the police were involved in these cases a total of 27 times. The critical importance of having security services present during violent situations was emphasized in several of the cases, as one nurse stated:

*“A patient that was psychotic and aggressive attacked and tried to strangle the doctor. A security guard, who was present, managed to prevent a serious incident.”*

Several incidents were described involving patients with no known history of violence or conditions that could explain uncontrolled violent behavior as described by a nurse who reported an incident involving a patient who appeared to be fully aware of his actions:

*“During the night shift I was punched in the throat by a patient while I was adjusting the blanket. I perceived it as a deliberate act.”*

In addition to staff being subjected to physical violence, the material also indicates that this often occurs in combination with psychological violence.

### 3.2. Psychological Violence

Psychological violence was described in 84 of the 247 reported cases. The different types of psychological violence and the number of reported incidents are summarized in Table 5. In several cases, multiple types of psychological violence were reported within a single case.

Of the 125 recorded incidents, 115 were carried out by patients, seven by relatives, one by both patient and relative, and two by other staff members.

In 37 cases, the healthcare personnel who have been subjected to psychological violence articulated the profound impact it has had on them. They reported experiencing stress, depression, insecurity, fear, and an inability to perform their duties as a result. Some incidents involved grave threats, including threats of murder, as well as posting staff information on social media. Although staff have overall reported relatively few threats related to social media, those that were reported tended to be of a very serious nature. One staff member wrote:

*“The patient becomes increasingly loud, shouting threats. The individual states an intention to spit on me, accuses me of having ruined their life, and says I will have to answer for it. The patient is visibly agitated and directs numerous insults toward me. It is mentioned that a picture of me has been posted on Facebook, along with a threat that someone will shoot me before the day is over. The patient showed me the post.”*

Our results consistently demonstrated that threats were frequently made but seldom carried out. In this specific case, however, the employee described not only psychological violence through threats, but also that the patient fulfilled the threats by posting the staff member’s picture and information on social media. In the report, the staff member further elaborated on the serious threats made by the patient:

*“I will kill you. Everyone working at this hospital should be killed, the admitting doctor and his children must be killed. I own this country, and you must leave. A high price has now been placed on your head, and this has been shared all over the world. You won’t live to see tomorrow; I will have you shot. I have been in prison before, so it’s worth it. I will send people to take your life.”*

In addition to patients threatening staff, there were also cases where staff reported unpleasant encounters and threats from the patient’s next of kin, as this staff member wrote:

*“A relative is very frustrated and speaks loudly in the corridor, expressing dissatisfaction with the hospital. This person states that a week ago, they wanted to throw a doctor out of the window. The relative emphasizes a willingness to go to great lengths for their family member and adds that if they are not heard, they may take unpleasant actions.”*

In our material, staff described aggressive and threatening behavior from relatives as unacceptable and as factors that heighten stress, anxiety, and fear. Another aspect described as psychologically demanding was when staff were threatened with weapons or weapon-like objects. In our material, we found that staff described threats involving various items, ranging from knives, forks, and razor blades to an electric tool, as detailed by this employee:

*“I was honestly terrified to be with the patient, but it is my job to ensure they receive necessary treatment. Fortunately, the patient had a security guard present, which made me feel somewhat safer. The patient was slamming doors, kicking, screaming, and speaking rudely. I experienced a physical reaction and was very scared every time there was a loud noise. Another security guard was assigned to the patient, but the outbursts continued. Then the patient became extremely angry and pulled out a large electric tool. It felt quite threatening. The security guards closed the door, and the police were called. The patient kicked and banged on the door, and the banging made my heart race and startled me every time.”*

Overall, the data indicated that such incidents result in staff experiencing a sense of danger due to distress. Despite facing physical and psychological threats, we observed descriptions of numerous accounts where staff diligently attended to the needs of patients and their relatives, even while fearing the outbursts of physical, psychological, and/or material violence.

### 3.3. Material Violence

Material violence was described in 28 of the cases, often in combination with psychological violence (16 cases) and physical violence (11 cases), as this incident illustrated:

*“The patient was unconscious in the ambulance but woke up before arriving at the hospital. The patient became very aggressive, requiring four nurses, one doctor, and a security guard to gain control. During the struggle, the patient punched a staff member, causing injury. Medication was administered, but the patient continued to make outbursts. The patient also tried to bite nurses, threw a glass at the chief physician, and damaged a PC screen.”*

The number of incidents are presented in Table 6. Of the 28 documented incidents related to material violence all incidents, except for one, were initiated by patients. The exception involved a staff member who, in an expression of anger, repeatedly slammed a door until it broke, resulting in anxiety among the remaining healthcare personnel. Our analysis identified instances of violent behavior directed towards material objects, including actions such as hitting and kicking furniture, walls, doors, medical equipment, and computer equipment, as well as throwing glass and destroying various items. Noise caused by material violence was also described.

Violence involving or directed at material objects or hospital property led to involvement of the security services in 24 cases and the police in eight cases. Material violence was the type of violent incident that most often caused healthcare personnel to alarm security personnel. Analysis of the descriptions revealed that incidents involving material violence were often perceived as particularly frightening by staff, as they generated an unpredictable environment when things were destroyed, banged on or thrown. In our material, we found several descriptions of how material violence and the noise from it, often in combination with physical and psychological violence, had a profound impact on the staff’s emotional and physical well-being. One staff member who was subjected to material violence described:

*“I would say that the consequences are that I did not (and do not) feel calm in any way. I still feel like I have my heart in my throat, feel my pulse in my chest… I couldn’t sleep last night, because every time I heard a sound, I got really scared. I jump every time I get surprised by, for example, a colleague coming around the corner, a sound I hear… I feel quite vulnerable and easily brought to tears. I think maybe it got a bit too much considering yesterday’s episode as well, that this has affected me so much.”*

This traumatic event left the person anxious, easily startled, and emotionally distressed, and the staff member also explained how a similar episode had played out the day before, adding to these feelings. In some cases, it appeared that both other patients and staff members became frightened when violent acts were directed at the hospital’s equipment or inventory, as one nurse described after she and her colleagues had been the victim of material violence:

*“Staff and patients were afraid; we called the security guard. The staff were crying and were unable to work until they recovered after a long time.”*

Causing fear and distress and the need for security intervention, we found that material violence had an emotional impact on staff members, leaving them anxious, easily startled and emotionally distressed.

### 3.4. Sexual Violence

While material violence causes significant distress, sexual violence poses an equally serious challenge, with profound implications for staff well-being. Sexual violence was described in ten cases in the dataset. All the acts were performed by patients. The police were involved in two cases, and security personnel in one of them. All cases were in combination with perceived physical and/or psychological violence. In four cases, the staff reported a feeling of mental strain. Descriptions included unsuitable physical or sexual contact, sexual harassment or inappropriate sexually related questions or suggestions, like one nurse reported:

*“A nursing student and I were tending to the patient. The patient was very unsteady and needed a lot of help. The patient became angry and tried to push me. Shortly after the incident, when the patient was back in bed, the patient asked me: ‘Have you ever been raped with a steel rod?”*

In addition to threatening and offensive comments, we also found descriptions of nurses being subjected to inappropriate touching, as well as patients touching their genitals in front of them. One nurse described:

*“I was alone with the patient. I was stuck in a corner when the patient started making inappropriate comments about my appearance and asked if I had a partner and where I lived. The patient had a catheter, and I was hanging the catheter bag on the bed when the patient grabbed my buttocks and squeezed them. The patient stood up again, removed their pants, and engaged in inappropriate behavior involving their private parts.”*

This form of unwanted sexual attention scared nurses and made them feel uneasy. All reported incidents happened when the staff were in a setting where they were trying to help patients maintain self-care or in an emergency.

### 3.5. Latent Violence

While sexual violence is overt and deeply impactful, latent violence operates in less visible ways but still influence staffs’ well-being. Only four incidents of latent violence were reported in our material, two of them overlapping with psychological violence. In these cases, we found descriptions of staff members modifying their actions to prevent potential tantrums from patients. Based on previous knowledge of a patient, one nurse described:

*“A patient with a history of aggressive behavior was admitted for monitoring. Upon arrival, the patient was awake and expressed a desire to go home but became aggressive when informed that this was not possible. The police were present. The doctor decided the patient should be treated, but the patient resisted it. Eventually, the on-call doctor decided that the patient could go home.”*

In this case, the patient did not commit any violence that generally would require presence from the police. However, due to the potential and latent possibility of violence, the staff made strategic choices, called the police and eventually discharged the patient.

In the material, we identified descriptions of latent violence in instances where patients’ actions signaled their potential for violent behavior, like in this scenario, where staff were required to retrieve a patient for involuntary commitment:

*“The patient has a known substance abuse problem and severe psychiatric illness. The substance abuse team met us upon arrival along with the police. They informed us that the patient always carries a knife and may act out. The patient verbally acts against healthcare personnel and is perceived as threatening towards relatives on site. Before the patient gets into our car, the patient takes out a large knife from their jacket and throws it on the ground in front of us.”*

In our material, we saw that the acts of latent violence were manifested through actions that implied aggression or threats, instilling fear and anxiety in those present.

## 4. Discussion

The analysis of reported incidents offers important insights into the types of violence experienced by staff in somatic healthcare. The results align with previous research [3,4,6,7,8,9,15] and underscore the widespread nature of workplace violence in healthcare settings. These results highlight the urgent need for effective interventions to reduce such incidents and protect healthcare personnel. Studies have shown that healthcare personnel often do not report workplace violence [13,18,19]. Fear of consequences, lack of knowledge, shame, and a non-supportive organizational culture are amongst the most common reasons for not reporting [13,18,19]. The absence of a shared understanding of what constitutes workplace violence can also contribute to the reluctance to report such incidents [13]. This can explain why some of the incidents in our material were reported by observing colleagues rather than by those directly subjected to them. This shows that without a shared understanding or a clear definition of what violence is, underreporting is more likely. Empirical evidence from Diez-Canseco suggests that the implementation of comprehensive and robust institutional guidelines addressing harassment may be associated with an increase in reported cases. Diez-Canseco claims that such trends should not be interpreted as a rise in the actual prevalence of harassment; rather, it can likely reflect improved employee awareness and a heightened sense of psychological safety [7]. Isdal [16] stresses that language serves as a central mechanism through which individuals make sense of their experiences and emotions. Labeling an incident as violence can be difficult, as it accentuates the emotional gravity of the experience while also taking a stance that affirms its seriousness. McLaughlin [18] argues that nurses often demonstrate insufficient understanding of which behaviors are deemed appropriate within a professional healthcare setting. Establishing a conceptual clarity regarding what constitutes violence and threats may therefore embolden healthcare personnel to report incidents.

Physical violence may be seen as the most fundamental form of violence, as it threatens bodily integrity and evokes fear of death [16]. Exposure to physical violence in the workplace may also adversely affect employees’ personal lives, manifesting as reduced sleep quality [10]. The predominance of physical violence within the incident reports aligns with findings from De Keyser and Bon [5], who observes high rates of physical aggression against healthcare staff in a systematic review. The comprehensive categorization of physical violence types in our study elucidates specific actions, such as hitting and kicking, contributing to a better understanding of the extent and nature of violent behaviors. Exposure to physical violence in the workplace may adversely affect employees’ personal lives, manifesting as reduced sleep quality. Furthermore, the frequent conjunction of physical and psychological violence underscores the complexity and multifaceted nature of violent incidents, necessitating multidimensional prevention and intervention strategies.

Psychological violence, as depicted in our results, is a significant concern, affecting 125 recorded incidents with various manifestations, including threats, shouting, and abusive language. These results are consistent with those of Ose et al. [15] and Kumar [11], who report a significant psychological burden on registered nurses as a result of workplace aggression. They also align with Jakobsson [12] and Schøsler et al. [10], who find that exposure to violence can lead to patterns of avoidance and absenteeism. Our results further demonstrate that such psychological impacts extend to significant fear, anxiety, stress, and compromised job performance among healthcare personnel. The most alarming threats were those indicating that the staff might be pursued and harmed outside of work, which is in line with the work of Jakobsson [12]. Humiliating, degrading, and devaluing characterizations that undermined healthcare personnel’s senses of dignity were described in our material. Shohani [3] describes such acts as cultural violence when they are directed toward the person’s ethnicity, race, language, religion, or place of origin. This form of violence reflects discriminatory attitudes and behaviors that undermine the dignity and safety of healthcare professionals from diverse backgrounds. Isdal [16] argues that psychological violence, through degrading language and attitudes, can be as harmful as physical violence, as it erodes the individual’s inner structure and dignity. As Cyberbullying has become an increasingly widespread issue in contemporary society [20,21], the incidents involving severe threats directed at staff and their families disseminated via social media highlight contemporary challenges in the digital age, requiring updated policies and comprehensive support systems for affected staff.

As demonstrated in our results, material violence in hospital settings poses a significant challenge, affecting both the physical surroundings and the emotional health of the staff. Isdal [16] asserts that the threshold for the use of material violence appears to be lower than for other types of violence. Material violence pertains to the exercise of influence and control by means of instilling fear or causing physical harm [16], and in the material, descriptions of emotional and physical repercussions were considerable. Staff members reported feelings of anxiety, fear, and distress following material violent incidents and noted that loud bangs were distressing. Our analysis reveals that episodes of material violence are frequently accompanied by both physiological and physical forms of violence. Examples include patients throwing objects at staff, attempts to destroy property, and noise resulting from violent actions. De Keyser and Boon [5] find that material violence frequently coincides with emotional dysregulation and is commonly associated with feelings of frustration or unmet psychological or situational needs. These incidents highlight the chaotic and unpredictable nature of material violence and the need for comprehensive safety measures that address multiple forms of violence simultaneously.

Material violence, defined as aggression directed toward property, was acknowledged within the broader framework of workplace violence, although it was not the central focus of the study. Such incidents frequently coincide with emotional dysregulation and are commonly associated with feelings of frustration or unmet psychological or situational needs.

The involvement of security personnel and police in managing violent incidents is significantly highlighted in the reported incidents. Security staff were involved in 65 of the physical violence cases, while police involvement was noted in 27 cases. This suggests the critical importance of having security measures and trained personnel present during high-risk situations to de-escalate potential violence effectively. As observed in several cases in our data, the presence of security can often avert serious physical harm, emphasizing the need for sufficient training for security forces in healthcare settings. Recurrent interactions with law enforcement can, however, result in heightened anxiety and stress among healthcare professionals, and it is suggested to implement evidence-based measures that minimize the need for law enforcement engagement with patients [22].

Another type of violence that frequently demanded interaction from law enforcement or security was sexual violence. In the cases described, performing intimate care tasks exposed healthcare personnel to situations involving patients’ sexual responses, thereby compromising the healthcare personnel’s sense of integrity and causing uneasiness. This result is in line with Friborg [23] and Adler [24] who find that all manifestations of sexual harassment demonstrate substantial associations with the deterioration of well-being among affected individuals [23]. According to Diez Canseco [7] and Quick [25], sexual harassment can result in a significantly higher prevalence of depressive symptoms in exposed individuals compared to individuals who have not been exposed. Sexual violence may be considered the most oppressive form of violence, as it targets the most private and vulnerable aspects of an individual [16]. Preventing these incidents must be a priority, as current strategies such as zero-tolerance harassment policies and training programs have consistently failed to protect victims from the psychological consequences, including depression [7,18]. This underscores the need for more effective, preventive approaches rather than relying solely on reactive measures.

There were a few incidents of latent violence described in the material. This is probably because latent violence goes unrecognized due to its concealed nature, making it challenging to identify. In our results, we discovered that incidents related to latent violence were often awaiting a catalyst to bring them to the forefront. The risk of violence shaped the behavior and choices of those exposed, leading to strategic and adaptive actions primarily aimed at preventing further victimization. This aligns with Isdal [16], who argues that violence is sustained by its mere possibility. This contrasts with the other forms of violence, which we found were more observable.

A detailed examination of the perpetrators reveals that patients carried out the majority of physical and psychological violence incidents, often described as having underlying conditions such as dementia, delirium, or substance abuse. This result aligns with the insights provided by several researchers [4,8,10], indicating that violent outbursts may frequently stem from patients’ medical conditions. These incidents illustrate the dual challenge healthcare professionals face: managing the medical needs of patients while simultaneously ensuring personal and peer safety. De Keyser and Boon [5] note that contextual factors frequently influence patient aggression and tend to arise in situations where expectations are not met and in environments characterized by stress-inducing conditions.

The data highlights several areas that require further investigation and action. Comprehensive policy reforms are necessary to provide healthcare professionals with effective support systems, including mental health support, legal aid, and structured training on managing aggressive behaviors [26,27]. Integrating technological advancements, such as threat assessment tools, and enhancing staff training in de-escalation techniques could be beneficial [26]. Fostering awareness and recognition of workplace violence in healthcare settings is essential [20], and policies must evolve to address digital threats robustly, providing clear guidelines and support mechanisms for staff facing online aggression.

### 4.1. Limitations and Strengths

In this study, 247 adverse events have been analyzed, providing a substantial dataset for identifying trends and drawing meaningful conclusions relevant to patient safety and nursing practice. The analysis was conducted meticulously and reviewed in collaboration with all authors, ensuring methodological soundness and enhancing the credibility of the results.

As the study was conducted in one hospital, the results may not be directly transferable to other institutions with different organizational structures or patient demographics. However, focusing on a single hospital allowed for a detailed understanding of local practices, systems, and reporting cultures, which strengthens the internal validity of the study. Including security healthcare personnel and altering the sampling plan may limit comparability with studies that focus exclusively on clinical staff. While this adjustment improved representativeness of high-risk groups, it introduces heterogeneity that should be considered when interpreting results.

The use of retrospective data may introduce biases related to the quality of reported events and completeness, potentially affecting the accuracy of the classification of the different types of violence [28]. Adverse events are known to be underreported in clinical settings particularly those perceived as minor events. This may have led to a dataset that does not fully capture the breadth of adverse events, potentially distorting the distribution and severity profile of the incidents analyzed. Consequently, the results may reflect only a portion of the actual safety challenges present in the hospital.

The lack of detailed patient level data constrained the ability to examine individual risk factors and outcomes linked to the reported adverse events. Furthermore, systemic contributors such as organizational structures or staffing conditions were not comprehensively analyzed, as these were beyond the primary scope of the study. Future research should consider exploring these dimensions to gain a more holistic understanding of the factors influencing adverse events. The manuscript adheres to the COREQ (COnsolidated criteria for Reporting Qualitative research) Checklist (Supplementary File) [29].

### 4.2. Implications for Future Research

Future research should focus on identifying effective intervention strategies tailored to specific types of violent behavior and the unique environment in healthcare settings.

Research should also examine effective strategies for supporting healthcare personnel after violent incidents, such as psychological follow-up and structured debriefing. Additionally, qualitative studies are needed to explore how healthcare professionals experience being subjected to violence, as this study provides limited insight into their personal perspectives.

## 5. Conclusions

The prevalence and complexity of violent incidents against healthcare professionals, as illustrated by the data and supported by relevant literature, underscore a need for actionable policies and research-driven interventions. By understanding the multifaceted nature of these violent acts and recognizing the psychological toll on healthcare personnel, we can better devise strategies to create safer, supportive, and more resilient healthcare environments. Clarifying the definition and scope of violence is imperative to ensure that healthcare professionals share a unified understanding and are adequately informed about the critical importance of reporting incidents when subjected to such experiences. Ensuring the safety of healthcare professionals is paramount to maintaining a functional and practical health system, especially in increasingly stressful and demanding health care landscapes. The results contribute to the theoretical expansion of Isdal’s framework [14] and offer insights for improving violence prevention and organizational adjustments. In doing so, the study aims to enhance the understanding of violence as a multifaceted phenomenon within a hospital context.

## Figures and Tables

**Table 1:** Different categories of violence according to Isdal.

Category	Description	Examples in a Hospital Context
Physical violence	Direct bodily harm that causes immediate physical and psychological effects.	Hitting, kicking, choking, biting.
Psychological violence	Acts that damage self-esteem, emotional stability, or mental health.	Threats, humiliation, manipulation, isolation.
Sexual violence	Unwanted sexual contact or abuse, involving both physical and psychological harm.	Inappropriate touching, sexual remarks, coercion.
Material violence	Damage to or control of physical objects/resources to instill fear or dominance.	Breaking equipment, destroying belongings.
Latent violence	Persistent underlying threat of violence, even if not enacted.	Verbal cues or behaviors creating fear of assault.

**Table 2:** Type of violence and number of reported incidents.

Type of Violence	Number of Reported Incidents
Physical violence	167
Sexual violence	10
Material violence	28
Latent violence	4
Psychological violence	125

**Table 3:** Overlapping acts of violence.

	Physical Violence	Sexual Violence	Material Violence	Latent Violence	Psychological Violence
Physical violence		4	11	0	41
Sexual violence	4		0	0	6
Material violence	11	0		0	16
Latent violence	0	0	0		2
Psychological violence	41	6	16	2	

**Table 4:** Types of physical violence and the number of reported incidents.

Type of Physical Violence	Number of Reported Incidents
Hitting staff	94
Kicking staff	54
Biting staff	22
Grabbing staff’s body parts	18
Spitting at staff	15
Pushing staff	11
Pulling staffs body	9
Twisting staff’s body	8
Attempt to strangle staff	6
Holding/restraining staff	6
Squeezing staff’s body	4
Headbutting staff	3

**Table 5:** Types of psychological violence and number of reported incidents.

Types of Psychological Violence	Number of Reported Incidents
Threatening statements	52
Threatening with violent acts	37
Shouting/yelling	25
Making unreasonable demands/complaints/statements	24
Abusive language	22
Racism	13
Destructive behavior	12
Name calling	11
Threating with a conventional weapon with the intent to hurt	11
Threating with non-conventional weapons/objects with the intent to hurt	8
Threatening staff in or with social media	7
Filming/taking photographs	7
Sexual harassment	3
Questioning/insulting	2
Unsuitable physical or sexual contact	2

**Table 6:** Types of material violence and the number of reported incidents.

Types of Material Violence	Number of Reported Incidents
Throwing objects at staff	6
Attempts to destroy property/items	17
Noise caused by material violence	6

## Data Availability

The data is not publicly available due to confidentiality.

## Supplementary Materials

The following supporting information can be downloaded at: https://www.mdpi.com/article/10.3390/nursrep16010005/s1, File S1: COREQ (COnsolidated criteria for REporting Qualitative research) Checklist [29].

## Public Involvement Statement

There is no public involvement in any aspect of this research.

## Guidelines and Standards Statement

This manuscript was drafted against the CoreQ checklist [29]. https://www.equator-network.org/, accessed on 15 November 2025.
